# Mixed oligosaccharides-induced changes in bacterial assembly during cucumber (*Cucumis sativus* L.) growth

**DOI:** 10.3389/fmicb.2023.1195096

**Published:** 2023-07-10

**Authors:** Qiushui Wang, Xin Zhou, Yue Liu, Yan Han, Jia Zuo, Jie Deng, Liyan Yuan, Lijuan Gao, Wenbo Bai

**Affiliations:** ^1^Institute of Analysis and Testing, Beijing Academy of Science and Technology (Beijing Center for Physical and Chemical Analysis), Beijing, China; ^2^State Key Laboratory of Mycology, Institute of Microbiology, Chinese Academy of Sciences, Beijing, China; ^3^Institute of Environment and Sustainable Development in Agriculture, Chinese Academy of Agricultural Sciences, Beijing, China

**Keywords:** mixed oligosaccharides, bacterial community, *Cucumis sativus* L., *Methylorubrum* spp., *Lechevalieria* spp.

## Abstract

The application of oligosaccharides can promote plant growth by increasing photosynthesis or inducing plant innate immunity. However, the mechanisms by which oligosaccharides affect bacterial community diversity and abundance remain unclear. In this study, a mixed oligosaccharide was applied to the growth of cucumbers. The findings of the present study suggest that the application of MixOS has significant effects on the bacterial communities in the phyllosphere, rhizosphere, and bulk soil of cucumber plants. The treatment with MixOS resulted in delayed senescence of leaves, well-developed roots, and higher fruit production. The bacterial diversity and composition varied among the different ecological niches, and MixOS application caused significant shifts in the bacterial microbiome composition, particularly in the phyllosphere. Moreover, mixed oligosaccharides increased the abundance of potential growth-promoting bacteria such as *Methylorubrum* spp. and *Lechevalieria* spp., and more zOTUs were shared between the WM and MixOS treatments. Furthermore, the bacterial co-occurrence network analysis suggested that the modularity of the phyllosphere networks was the highest among all samples. The bacterial co-occurrence networks were altered because of the application of MixOS, indicating a greater complexity of the bacterial interactions in the rhizosphere and bulk soil. These findings suggest that mixed oligosaccharides has the potential to improve plant growth and yield by modulating the bacterial communities within and outside the plants and could provide a theoretical basis for future agricultural production.

## 1. Introduction

Cucumber (*Cucumis sativus* L.) is one of the most valuable greenhouse vegetables in China that is cultivated extensively due to its increasing market demand (Li et al., 2021). However, the excessive use of pesticides and fertilizers for achieving higher yields has led to several detrimental effects such as soil fertility erosion, loss of biodiversity, nutrient and pesticide runoff, and negative impacts on health and the environment. Therefore, there is an urgent need to reduce fertilizer dependency in order to mitigate the negative impacts of agriculture on the surrounding environment. This has led to an increased demand for plant growth regulators in crop production and agriculture practice (Jin et al., 2019; Li et al., 2021).

Chitosan oligosaccharide (COS), which is derived from the enzymatic hydrolysis of chitosan, has recently gained attention for its various application such as a health food (Chantarasataporn et al., 2014; Guarnizo et al., 2020), plant growth stimulator, feed additive, antimicrobial, and drugs (Li and Zhu, 2013; Chatelain et al., 2014; Swiatkiewicz et al., 2015; Jia et al., 2019; Chang et al., 2020; Guarnizo et al., 2020; Tao et al., 2022; Tzeng et al., 2022). In agriculture setting, COS has been found to effectively induce plant innate immunity, improve tolerance to adverse environmental stresses, and promote plant growth. For instance, it increasees photosynthesis in *Dendrobium* orchids by increasing their chlorophyll content and improving the chilling and salt stress tolerance of wheat seedlings (Limpanavech et al., 2008; Zhang et al., 2017; Zou et al., 2017). Overall, oligosaccharides function as growth promoters and bactericides, suppress pathogen growth, and preserve plant yield and quality (Mukhtar Ahmed et al., 2020). Plants have a long history of coevolution with microorganisms, which perform several crucial ecological functions on the surfaces of roots and leaves, as well as within plant tissues (Chen et al., 2020; Zhou F. et al., 2022; Zhou et al., 2022a). For example, the *Colletotrichum tofieldiae* and fungal species in AMF group have provided helps to host plants in absorbing phosphorus (Hiruma et al., 2016). Therefore, the application of oligosaccharide could induce changes in bacterial assembly during plant growth.

In this study, we hypothesized that bacterial communities shifted owing to the application of KROPICO, a type of mixed oligosaccharide produced by Showa Denko and that the altered bacterial communities could play an important role in the growth process of cucumbers. Based on this hypothesis, a high-throughput sequencing technique was used to analyze the changes in bacterial composition and diversity of samples from different ecological niches, including the phyllosphere, rhizosphere, and bulk soil, during cucumber growth in a greenhouse. This study provides a theoretical basis for explaining the beneficial effects of mixed oligosaccharides on bacterial community assembly.

## 2. Materials and methods

### 2.1. Experimental design and sampling

All the samples were collected from the same cucumber greenhouse in Tianjin, China (Bao Di, 40.05 N, 117.27 °E). The cucumber cultivar used in this study was ‘Jinlv4’ which is the same as that used by local farmers. Three different treatment groups were designed with different management strategies, namely water mimic (WM), mixed oligosaccharide (MixOS), and regular treatment (RT) groups. In the WM treatment, the roots were dipped in tap water before being transplanted into the greenhouse. In the following seedling stage, all the leaves in WM group were sprayed with tap water eight times (1,100 kg∙hm^−2^∙time^−1^, once a week). During the entire experimental period, all the cucumber plants in the WM group were watered and fertilized in the same manner as the plants in the RT group, and insecticides were applied. In the MixOS treatment, the roots were dipped in 1000-fold diluted KROPICO (Showa Denko, Japan) before being transplanted into the greenhouse. In the seedling stage, the leaves in the MixOS group were sprayed with diluted KROPICO eight times (1,100 kg∙hm^−2^∙time^−1^, once a week). During the entire experimental period, all the cucumber plants in the MixOS group were watered and fertilized in the same manner as the plants in the RT group, and insecticides were applied. In the RT treatment, cucumbers were planted based on the manager’s recommendations, including watering, normal fertilization, and supplementation with insecticides and microbicides. The method used to determine the chlorophyll content, enzyme activities, and yield were described in Supplementary Methods S2, S3, respectively.

Three batches of samples were collected during the cucumber growth period, including the full flowering stage (FFL), full fruit stage (FFR), and seedling pulling stage (SPU). Each treatment group was replicated biologically with a minimum of six cucumber plants (ranging from six to eight). All the cucumber plants together with the bulk soil were transported back to the laboratory in dry ice and stored at −80°C for DNA extraction. During the cucumber growth period, all the mature cucumbers were harvested from the plants, weighed, and counted to determine the yield from each group.

### 2.2. DNA extraction and amplicon sequencing

To prepare the samples for high-throughput sequencing, cucumber roots were manually shaken to remove bulk soil attached to the roots, leaving 1 mm of soil for the following process. The roots were then washed, filtered, and centrifuged as outlined in the optimized protocol described by Edwards et al. (2015) to produce rhizosphere pellets. Those precipitated pellets were components of the rhizosphere. Genomic DNA of all samples, including leaf (~1 g), rhizosphere component, and bulk soil (~1 g), were extracted using the FastDNA^®^ Spin Kit (MP Biomedicals, Solon, OH, United States). DNA quality and concentration were assessed using 1.0% agarose gel electrophoresis and a NanoDrop^®^ ND-2000 spectrophotometer (Thermo Scientific Inc., United States), respectively, before further use. To profile the bacterial communities, we amplified the hypervariable region V3-V4 of the bacterial 16S rRNA using the primer pairs 338F (5’-ACTCCTACGGGAGGCAGCAG-3′) and 806R (5’-GGACTACHVGGGTWTCTAAT-3′) (see Supplementary Method S1 for details). In brief, the PCR amplifications were performed with 20 ng of template DNA, 0.4 μM forward and reverse primers, and 12.5 μL HiFi HotStart Ready mix (Kapa Biosystems) in 25 μL volume. The conditions of PCR reaction were as follows: denatured for 10 min at 95°C; 25 cycles of 95°C denaturation for 30 s; 55°C annealing for 30 s and 72°C extension for 60 s; final extension of 72°C for 10 min and hold at 4°C. The non-template control PCR reaction was set up for each batch of PCR reactions, and only products with no electrophoretic band in negative control were kept for the next step experiments. The final amplicon libraries were used for paired-end sequencing on an Illumina MiSeq PE300 platform (Illumina, San Diego, CA, United States).

### 2.3. Analysis of amplicon sequencing data

Bacterial 16S rRNA raw sequences were processed using USEARCH v11 (Edgar, 2018) and EasyAmplicon pipeline v1.18 (Liu et al., 2023). The primer sequences and low-quality reads were trimmed using the-fastx_filter command, and paired reads were merged into a single sequence using the-fastq_mergepairs command in USEARCH v11. All the correct biological reads were dereplicated using -fastx_uniques command and denoising into zero-radius operational taxonomic units (zOTUs) with 100% similarity using the unoise3 (Edgar, 2016) command in USEARCH v11. All the chimeric sequences were defined and removed using the uchime_ref command against the SILVA (v138) database. The taxonomy of the bacterial zOTUs was classified using the USEARCH sintax algorithm against the SILVA (v138) database (Quast et al., 2013). All zOTUs assigned to plastids and non-bacteria assigned to plants or protists were removed using in-house scripts developed in the EasyAmplicon package (Liu et al., 2023). Bacterial α- and β-diversity were calculated using the vegan v2.6–4 package (Oksanen et al., 2007), and the plots were generated using the ggplot2 v3.4.1 package (Wickham, 2016).

### 2.4. Statistical analysis

Before conducting diversity and statistical analysis, the zOTU table was normalized to the lowest read number of bacterial samples which was 27,272 reads per sample. To determine the species diversity of the treatments, Shannon’s entropy and richness indices were utilized. Significant differences between groups were calculated using analysis of variance (ANOVA) and Tukey’s honest significant difference test. The bacterial communities were assessed using Bray-Curtis distances, and the differences in community composition were examined through two-way nested analysis of similarity (ANOSIM) and multivariate permutation analysis of variance (PERMANOVA). To investigate zOTUs enriched in different treatments, an analysis of ternary plots was conducted in R using average relative abundances that were transformed by log2. The co-occurrence network analysis was conducted using the bacterial zOTUs with relative abundances greater than 0.1%. The non-parametric Spearman correlation algorithm was used to calculate the topological properties and reconstruct co-occurrence network patterns. The Spearman’s correlation coefficient *ρ* > 0.70 and the significant *p* < 0.05 were regarded as a robust co-occurrence network (Benjamini–Hochberg adjusted) (Zhou et al., 2022a). Spearman’s rank correlation analysis was employed to calculate the correlation relationships between enriched bacterial taxa and KEGG pathway functions. Only correlations with |*r*| > 0.7 and FDR < 0.01 were shown with ^*^ in the heatmap plots.

## 3. Results

### 3.1. Mixos delayed plants senescence and increased its production

**Figure 1:**
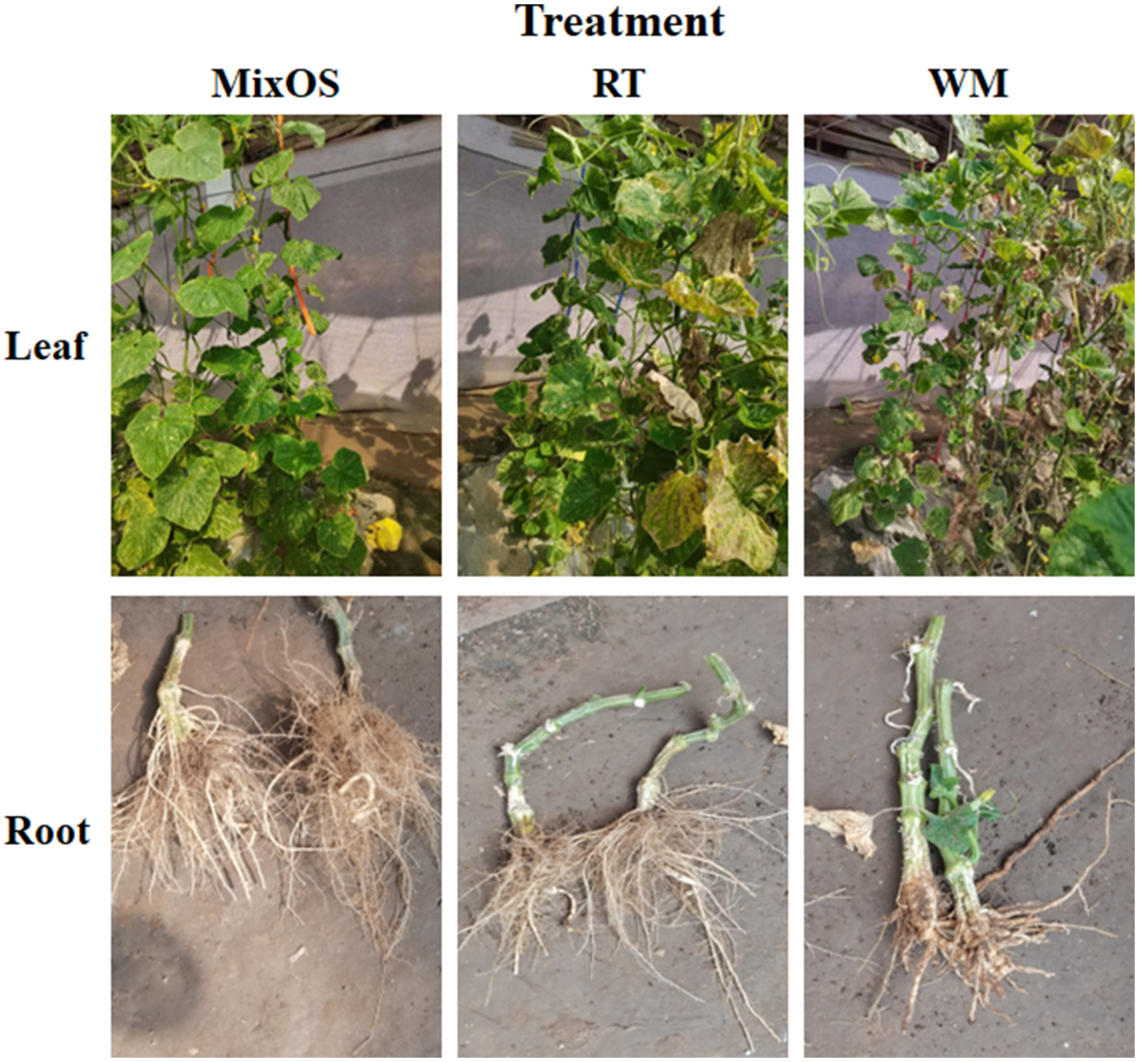
Plant phenotypes of three different treatment groups. Treatment RT (middle panels) and WM (right panels) are controls. MixOS (left panels) is the experimental group treated with oligosaccharides complex. Leaves (top panels) and roots (bottom panels) are presented, respectively.

In the present study, treatment with MixOS resulted in delayed senescence compared to the untreated groups. As depicted in Figure 1, the leaves in the MixOS group remained fresh and intact, while the leaves in the RT and WM groups were withered and yellow. Most of the leaves in the WM group were also kraurotic and the related chlorophyll and enzyme activity, have been added in Supplementary Table S1. Although differences were observed in the root phenotypes, cucumber roots from the MixOS group were well-developed with numerous branched roots, most of which were longer than those in the other two groups (yield per plant, Supplementary Table S2). Conversely, the roots from the RT and WM groups were weaker than those in the MixOS group. These phenotypic differences indicated that MixOS affected both leaves and roots, and suggests interactions between bacterial communities within and outside the plants.

To determine whether MixOS had an impact on cucumber fruit, we quantified the monthly production and total yield during the last growth period (Supplementary Figure S1). Based on the production calculations, the MixOS group had substantially higher production at every sampling time, and this promoting effect persisted until the final sampling point (Supplementary Figure S1A). Additionally, we observed an increase in total yield in the MixOS group (Supplementary Figure S1B) which suggests greater economic benefits in the future.

### 3.2. Microbial diversities and compositions are different in bacteria with MixOS treated

**Figure 2:**
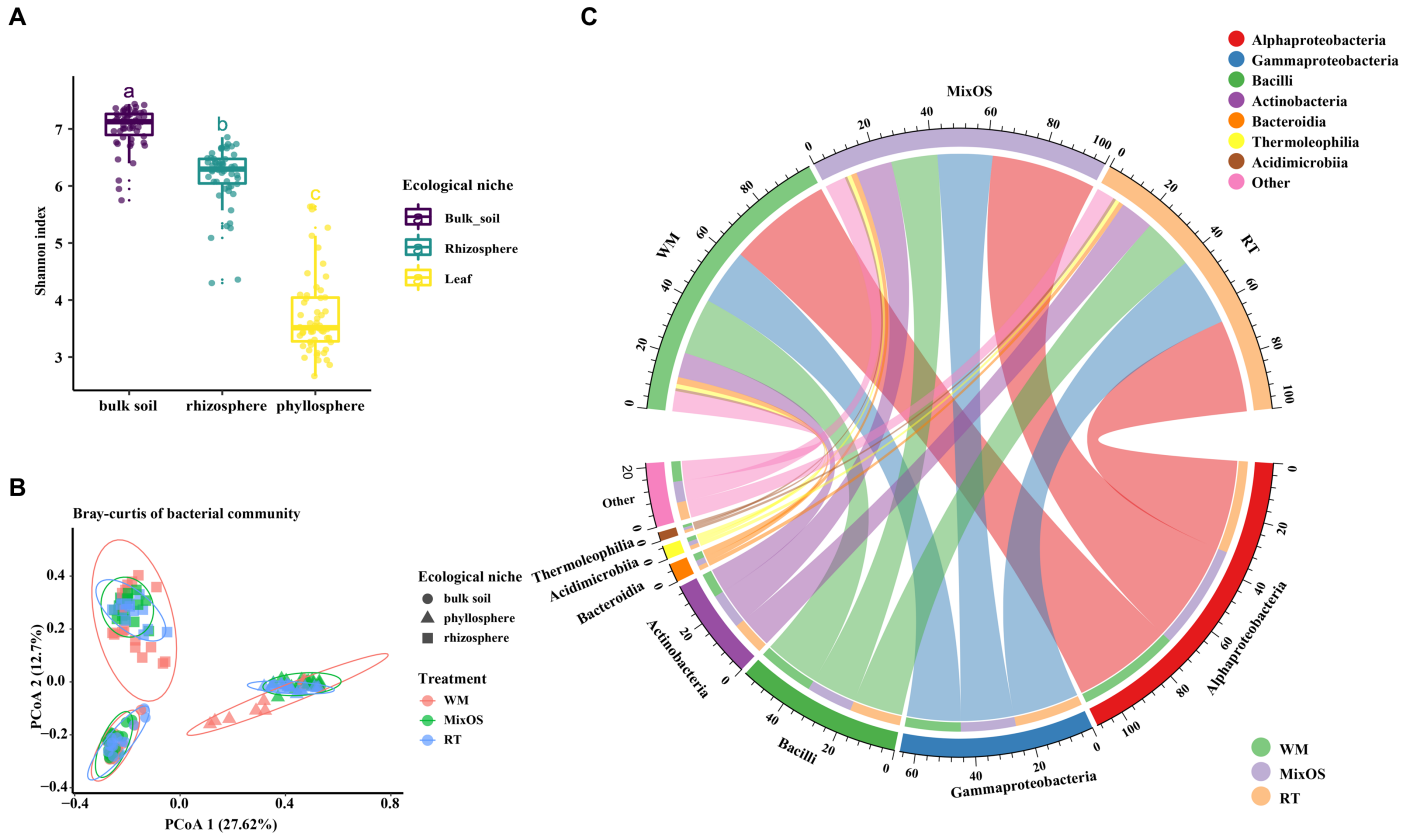
**(A)**, Shannon index values of bacterial diversity across different ecological niches. **(B)**, principal coordinates analysis of bacterial communities based on Bray–Curtis distance dissimilarities. **(C)**, The relative abundance of the most abundant bacterial taxa at class level from three different treatments.

**Figure 3:**
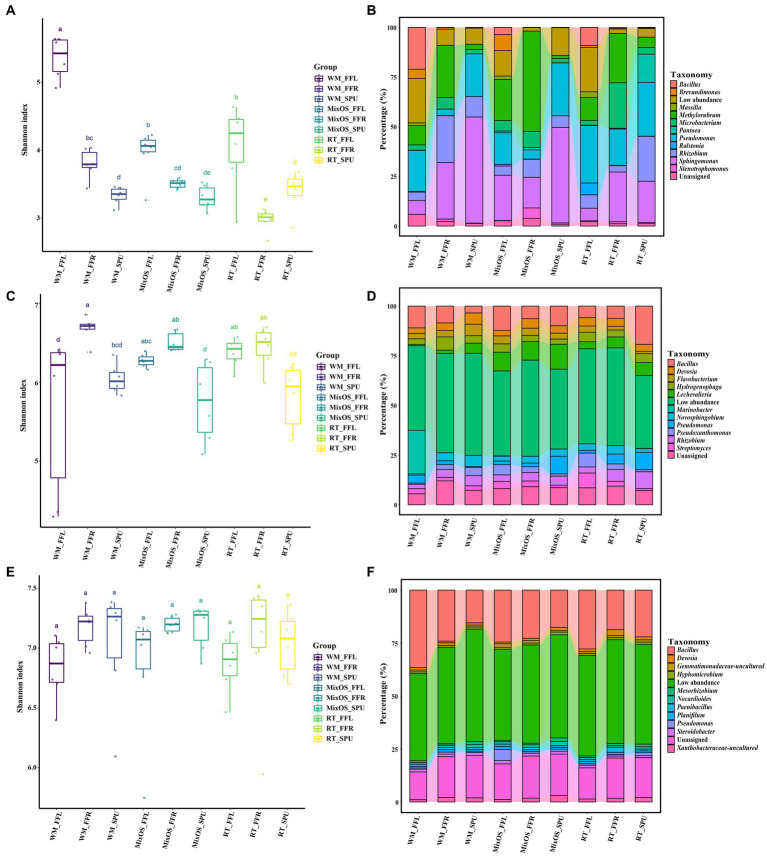
The difference in α diversity and bacterial composition among all treatment groups. Bacterial diversities were shown with Shannon index in phyllosphere **(A)**, rhizosphere **(C)** and bulk soil **(E)**. The lines inside boxes represent the median at 95% confidence. The significant differences in the diversity indices between different continents were annotated with different lower-case letters (*p* < 0.05). Relative abundance of the most dominant bacterial genera from the phyllosphere **(B)**, rhizosphere **(D)**, and bulk soil **(F)** communities.

To determine the bacteria diversity and composition among all samples, a comparison analysis of α and β diversity was performed. Bacterial richness varied among different ecological niches. Pairwise comparisons of α index values revealed that bacterial communities in bulk soil had the highest bacterial diversity, whereas the phyllosphere bacterial communities exhibited the lowest bacterial diversity (*p* < 0.05, Turkey HSD test) (Figure 2A). Bacterial richness significantly differed among the different treatments (RT, MixOS, and WM) in the phyllosphere communities (Figure 3A). However, differences among RT, MixOS, and WM treatments in the rhizosphere and bulk soil communities were not significant in terms of the Shannon index (Figures 3C,E). To evaluate dissimilarities in bacterial communities among samples from different groups, the Bray–Curtis dissimilarity matrix was employed. PCoA plots demonstrated that axis 1 (Pco1) explained 27.62% of the total variation, while axis 2 (Pco2) explained 12.7%. With respect to bacterial communities, three distinct groups were identified: bulk soil, rhizosphere, and phyllosphere (ADONIS, *p* < 0.01). The bacterial communities among the different treatments (RT, MixOS, and WM) in the bulk soil and rhizosphere groups were grouped on Axes 1 and 2, respectively. However, minor variations in the bacterial communities among the different treatments (RT, MixOS, and WM) were observed in the phyllosphere group, indicating pronounced effects on phyllosphere bacterial communities (Figure 2B). The PCoA analysis results for each nich were also presented in Supplementary Figure S2.

### 3.3. MixOS changes bacterial microbiome composition

To more effectively understand the effect of MixOS on the microbiome across the phyllosphere, rhizosphere, and bulk soil, weinvestigated microbial shifts among different treatments and the temporal dynamics of the microbiome in each treatment. At the class level, the Alphaproteobacteria (26.8%), Bacillus (16.2%), Gammaproteobacteria (15.7%), and Actinobacteria (12.0%) class were dominant, followed by Bacteroidia (4.8%), Thermoleophilia (3.0%), and Acidimicrobiia (2.2%) (Figure 2C). At the genus level, the most abundant bacteria in the phyllosphere were*Bacillus*, *Brevundimonas*, *Massilia*, *Methylorubrum*, *Microbacterium*, *Pantoea*, *Pseudomonas*, *Ralstonia*, *Rhizobium*, *Sphingomonas*, and *Strenotrophomonas* (Figure 3B). The proportion of *Methylorubrum* was notably higher with MixOS application compared to the RT and WM treatments. All the bacteria in the phyllosphere showed a dynamic process of change within each treatment group, while the trends were similar among the three treatment groups.

Bacterial communities in the rhizosphere and bulk soil remained relatively stable across different treatments and sampling periods. The most abundant genera in the rhizosphere were *Bacillus*, *Devosia*, *Flavobacterium*, *Hydrogenophaga*, *Lechevalieria*, *Marinobacter*, *Novosphingobium*, *Pseudomonas*, *Pseudoxanthomonas*, *Rhizobium*, and *Streptomyces* (Figure 3D). A comparative analysis revealed that MixOS application caused the *Lechevalieria* community to shift across all sampling periods. The dominant genera in the bulk soil communities were *Bacillus*, *Devosia*, *Gemmatimonadaceae*, *Hyhomicrobium*, *Mesorhizobium*, *Nocardioides*, *Paenibacillus*, *Planifilum*, *Pseudomonas*, *Steroidobacter*, and *Xanthobacteraceae* (Figure 3F). Genera with low abundance were highly represented in the rhizosphere and bulk soil bacterial communities, indicating higher bacterial diversity in the rhizosphere than those of phyllosphere.

### 3.4. Comparison among different treatment groups

**Figure 4:**
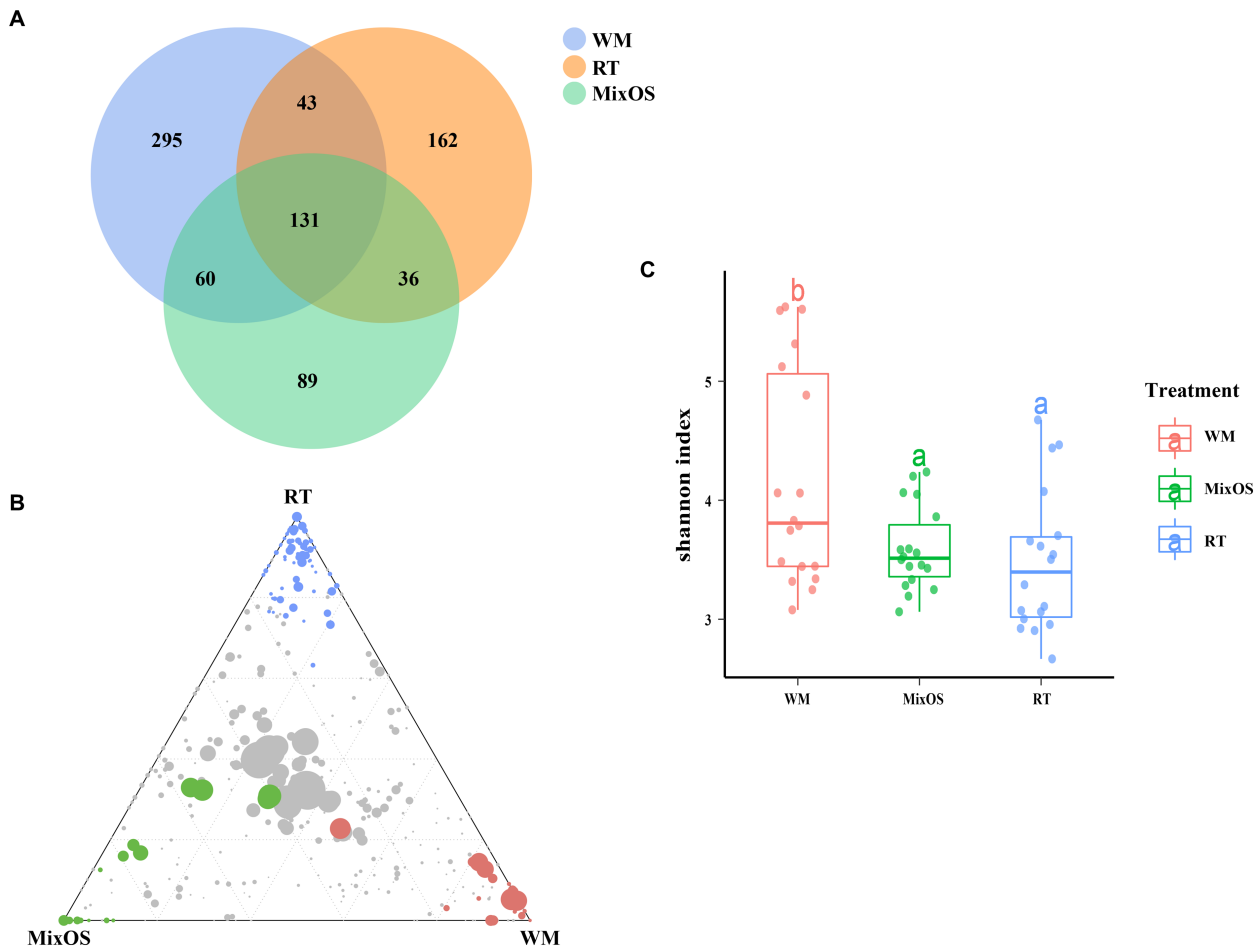
**(A)** Veen diagrams of phyllosphere microbiota from different treatments. The ternary plot showed significantly enriched phyllosphere bacterial zOTUs **(B)** in the WM group (read filled circles), RT group (blue filled circles), and MixOS group (green filled circles), respectively. The grey dots in the center of the ternary plots represent non-significant zOTUs shared by all treatment groups. **(C)** Shannon index values of phyllosphere bacterial diversity across different treatments. The Shannon index values are colored by different treatments, the line within the box represents the median and bottom, and the top boundaries of the box indicate the 75th and 25th percentile, respectively.

The Venn diagrams Figure 4A and Supplementary Figures S3A,B indicated that the treatment groups shared 131 phyllosphere bacterial zOTUs, 829 rhizosphere bacterial zOTUs, and 1,436 bulk soil bacterial zOTUs. More zOTUs were shared between the WM and MixOS treatments compared to the RT treatment in the phyllosphere bacterial communities. Specifically, 60 zOTUs were shared between the WM and MixOS treatments The results have shown that phyllosphere bacterial communities under shared (Figure 4A). Almost the same number of rhizosphere bacterial zOTUs (WM vs. MixOS:179, MixOS vs. RT:183, and WM vs. RT:185) were shared between the WM, MixOS, and RT treatments (Supplementary Figure S3A). Similar to phyllosphere bacterial zOTUs, more bulk soil bacterial zOTUs were shared between the WM and MixOS treatments (Supplementary Figure S3B).

The ternary plot showed that specific zOTUs associated with each treatment corresponded to points at the corners of each group (Figure 4B, Supplementary Figures S3C,D). The MixOS treatment group significantly enriched abundant of phyllosphere bacterial zOTUs which belongs to the genera of *Microbacterium*, *Sphingomonas*, and *Rhizobium* (Figure 4B). In contrast, a relatively small number of phyllosphere bacterial genera including *Pantoea*, *Pseudomonas* and *Labedella* with lower abundance were enriched in the RT treatment (Figure 4B). The bacterial diversity of the phyllosphere in the RT treatment significantly differed from the phyllosphere microbiota in the WM treatment (Figure 4C). For example, the bacterial zOTUs enriched in the WM treatment were mainly belong to genera like *Bacillus*, *Brevundimonas*, *Curtobacterium*, *Erwinia*, *Paucisalibacillus*, *Methylobacterium-Methylorubrum*, and other low abundant bacterial genera. However, there were no significant rhizosphere and bulk soil microbiota showed no significant differences among treatments (Supplementary Figures S3C–F). The Spearman correlation coefficients was used to calculate the correlation between enriched bacteria and KEGG pathways. The result showed that the bacterial genera of *Mesorhizobium* (|*r*| > 0.7, *p* < 0.05), *Shinella* (|*r*| > 0.7, *p* < 0.05), *Nocardiopsis* (|*r*| > 0.7, *p* < 0.05), *Pseudonocardia* (|*r*| > 0.7, *p* < 0.05), *Pseudonocardia* (|*r*| > 0.7, *p* < 0.05), and *Luteimonas* (|*r*| > 0.7, *p* < 0.05) had very similar positive correlations patterns with most KEGG properties, such as Carbon fixation pathways in prokaryotes, Fatty acid metabolism, Phosphotransferase, Polyketide sugar unit biosynthesis, Ion channels (Supplementary Figure S4, Supplementary Table S3). For the bacterial communities of cucumber leaf, the enriched *Sphingomonas* spp. were positively correlated with Carbohydrate metabolism, Glycosaminoglycan degradation, Pertussis, and Pores ion channels (Supplementary Figure S5, Supplementary Table S4). *Microbacterium* spp. were positively correlated with Progesterone−mediated oocyte maturation, Biosynthesis and biodegradation of secondary metabolites, and Glutamatergic synapse. *Methylobacterium − Methylorubrum* spp. were positive correlated with Stilbenoid, diarylheptanoid, and gingerol biosynthesis (Supplementary Figure S5, Supplementary Table S4).

### 3.5. Bacterial co-occurrence networks altered because of the application of MixOS

**Figure 5:**
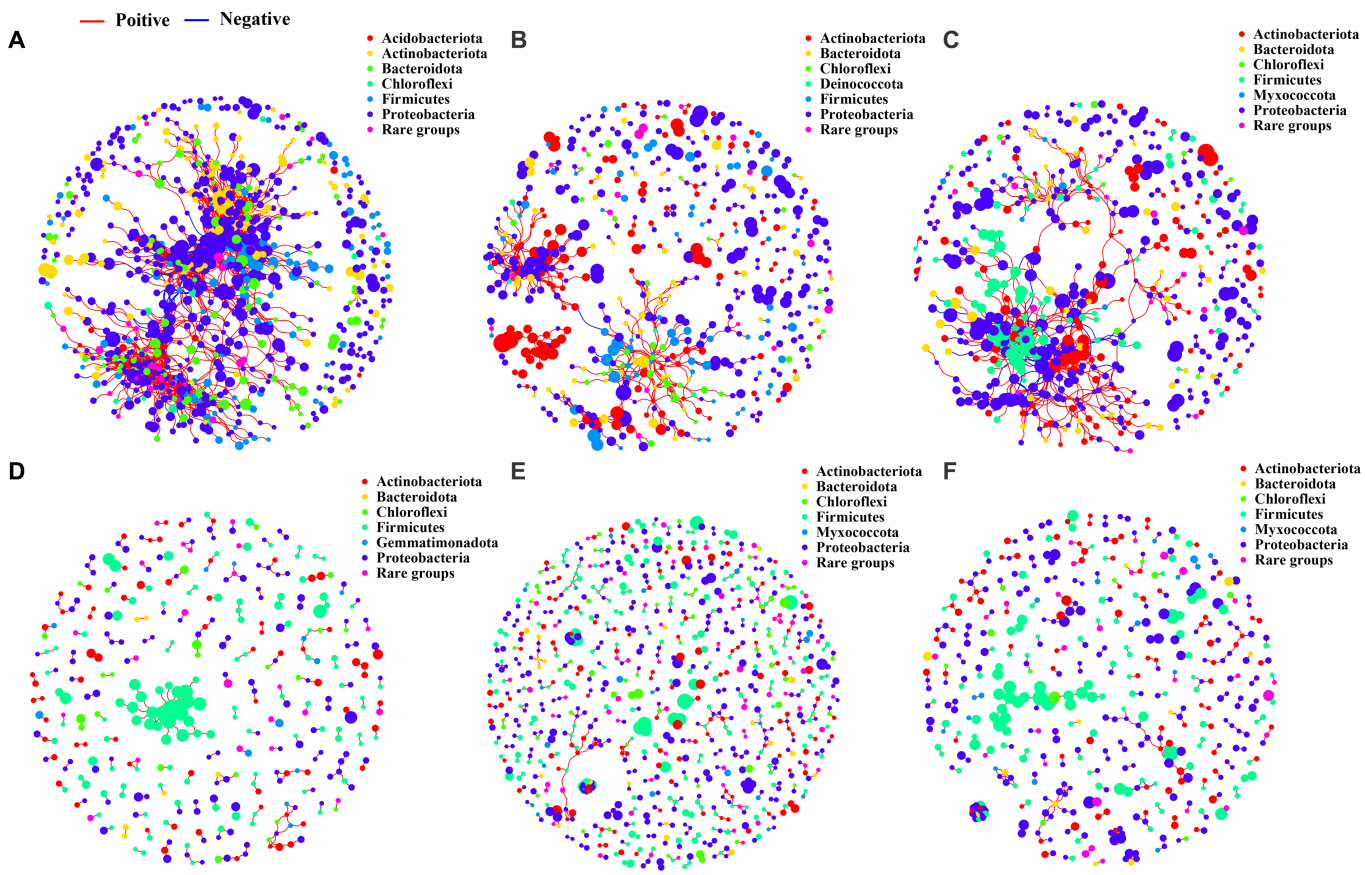
Bacterial co-occurrence networks. **(A)**, networks of rhizosphere bacterial communities in the WM treatment. **(B)**, networks of rhizosphere bacterial communities in the MixOS treatment. **(C)**, networks of rhizosphere bacterial communities in the RT treatment. **(D)**, networks of bulk soil bacterial communities in the WM treatment. **(E)**, networks of bulk soil bacterial communities in the MixOS treatment. **(F)**, networks of bulk soil bacterial communities in the RT treatment.

Based on the bacterial co-occurrence network analysis, the degree and closeness centralities of the networks were found to be significantly higher in the rhizosphere (Figures 5A–C; Supplementary Table S5) and bulk soil (Figures 5D–F; Supplementary Table S5), compared to the phyllosphere (Supplementary Figures S6A–C; Supplementary Table S5). The bacterial co-occurrence networks in the rhizosphere and bulk soils were observed to be less dense and more isolated compared to those in the phyllosphere. The greater average degree and network connectivity in the rhizosphere and bulk soils indicate the greater complexity of the bacterial co-occurrence networks in these niches. The modularity of the phyllosphere bacterial co-occurrence networks was found to be much higher than that of the rhizosphere and bulk soil. Additionally, the modularity of the networks in the rhizosphere was observed to be higher than that in the bulk soil.

Taxonomic assignments showed that the bacterial networks in all three different ecological niches were primarily composed of the phyla Acidobacteria, Actinobacteria, Bacteroidetes, Chloroflexi, Firmicutes, and Proteobacteria (Figure 5; Supplementary Figure S6). However, notable differences were observed between the MixOS and RT treatments of the phyllosphere bacterial networks, including the absence of some primary phyla absent and the presence of two additional phyla, Fusobacteriota (Supplementary Figure S6B), and Verrucomicrobiota (Supplementary Figure S6C). Application of MixOS treatment to the rhizosphere resulted in a significant decrease in the connectedness and centrality of the nodes, indicating fewer modular bacterial networks in this treatment (Figure 5B).

## 4. Discussion

Microorganisms exert a crucial influence on regulating soil fertility (Chen et al., 2018), and enhancing plant fitness (Zhou et al., 2022a), which has a close correlation with plant performance. In this study, the amplicon approach was used to investigate the effects of a mixture of oligosaccharides (MixOS) and KROPICO on the cucumber microbiome. Our found reveal that MixOS effectively delayed cucumber leaf senescence and significantly increased cucumber production. These observations align with previous studies that demonstrated similar beneficial effects of chitosan and oligochitosan treatments in delaying the ripening and senescence of peach fruits by regulating antioxidant enzymes (Ma et al., 2013).

Moreover, the application of MixOS had significant effects on bacterial diversity and community composition, particularly in the phyllosphere microbiota. Our study revealed that bacterial communities in bulk soil exhibited the highest bacterial diversity, while those in the phyllosphere bacterial communities displayed the lowest bacterial diversity. Futhermore, the diversity indices of the phyllosphere bacterial communities showed a decreasing trend in the MixOS treatment. These findings are consistent with previous research indicating that chitosan and its oligosaccharides can inhibit a wide range of bacterial plant pathogens(Liu et al., 2007; Rabea and Steurbaut, 2010; Badawy et al., 2014; Dodgson and Dodgson, 2017). The dominant bacteria in this study belonged to the phyla Proteobacteria and Bacteroidetes, which have been suggested to exhibit copitrophic behaviour (Ezazi et al., 2021). Interestingly, we observed an increase in the proportion of *Methylorubrum* spp.following MixOS application. *Methylorubrum* spp., reclassified from the genus *Methylobacterium* (Green and Ardley, 2018), produce growth-promoting metabolites by consuming methanol secreted by plants (Sanjenbam et al., 2022; Jin et al., 2023). *Methylorubrum* predominates during the flowering stage of soybean shoots due to the difference in nitrogen-fixing activity and concentration of nitrogen compounds in the xylem sap before and after the flowering stage (Hara et al., 2019; Zhou et al., 2022b). Our results also proved that *Methylorubrum* were significantly positively Carbon fixation pathways in Fatty acid, Phosphorus, Polyketide sugar, and Iron ion, which could promote the growth of cucumber plants as well as disease suppression. It has been suggested that the shify in nitrogen conditions caused by MixOS utilisation led to *Methylorubrum* dominance. Additionally, the application of MixOS caused a shift in the *Lechevalieria* community across all the sampling periods in the rhizosphere. *Lechevalieria* spp. have the potential to act as effective biological control strategies for plant production systems (Cuesta et al., 2012). Overall, the application of MixOS increased the abundance of *Methylorubrum* spp. and *Lechevalieria* spp. (Jin et al., 2023), indicating that MixOS stimulated potential growth-promoting microorganisms in the phyllosphere and rhizosphere, respectively.

Although MixOS application did not significantly affect the bacterial composition in the rhizosphere or bulk soil (Supplementary Figures S7B,C), it had a noteworthy impact on phyllosphere bacterial communities (Supplementary Figure S7A). This finding suggests that MixOS play a crucial role in phyllosphere bacterial communities. According to differential abundance analysis of the community composition, two phyllosphere bacterial classes, *Bacteroidia* and *Bacilli*, were depleted in the MixOS treatment compared to the WM treatment. Meanwhile, a substantial proportion of members of the classes *Actinobacteria*, *Alphaproteobacteria* and *Gammaproteobacteria* were depleted in the MixOS treatment, while another small proportion was enriched in MixOS, including *Methylorubrum* spp. belonging to the class *Alphaproteobacteria*. These results indicate that MixOS exhibits antibacterial activity against some bacteria (Mukhtar Ahmed et al., 2020). Furthermore, network modularity and cooperative and competitive interactions among microbial species can influence community stability (Gao et al., 2021). The lower modularity in the MixOS treatment among all ecological niches in this study may worsen the destabilising effect due to the higher prevalence of cross-module correlations among taxa (Grilli et al., 2016; Hernandez et al., 2021). The modularity of the phyllosphere bacterial co-occurrence networks was the highest among all samples, indicating the most positive effect on phyllosphere stability compared to the rhizosphere and bulk soil. Together, our findings suggest that MixOS could enrich beneficial bacterial taxa and play relevant roles in the growth-promoting and disease suppression of cucumber plants. Based on our findings, we suggest applying MixOS in the agriculture practice of cucumber plants. Moreover, the potential beneficial species could be verified by culture-based approaches. Beyond the scope of the current study, future studies could try to isolate these potential beneficial taxa which were enriched by MixOS application and evaluate the consistency of growth-promoting and disease suppression ability *in vivo*. Our study provides baseline information for the application of MixOS in the cucumber agriculture practice of plants.

## 5. Conclusion

This study has revealed that the ecological niche had the most significant impact on the assembly of the bacterial microbiome, followed by the treatment mode. MixOS had a considerable effect on the diversity and composition of the phyllosphere microbiota, stimulating growth-promoting microorganisms like *Methylorubrum* spp. and *Lechevalieria* spp., in the phyllosphere and rhizosphere, respectively. Compared with the RT treatment, MixOS played a more critical role in altering the bacterial co-occurrence network, reducing the connectedness and centrality of nodes. This study has significantly enhanced our understanding of bacterial microbiome assembly through the MixOS application and offers the potential for using mixed oligosaccharides to promote plant growth and sustainable agricultural production. These findings provide a theoretical basis for exploring the mechanisms by which MixOS enhances cucumber growth and guiding future agricultural production.

## Data availability statement

The datasets presented in this study can be found in online repositories. The names of the repository/repositories and accession number(s) can be found in the article/Supplementary material.

## Author contributions

QW, LG, and WB designed the experiments. QW, YL, YH, JD, LY, and JZ performed the experiments. QW, XZ, YH, and LG analyzed the data. QW and XZ wrote the manuscript. LG and WB revised the manuscript. All authors contributed to the article and approved the submitted version.

## Funding

This work was supported by the National Key Research and Development Program of China (2019YFE0197100).

## Conflict of interest

The authors declare that the research was conducted in the absence of any commercial or financial relationships that could be construed as a potential conflict of interest.

## Publisher’s note

All claims expressed in this article are solely those of the authors and do not necessarily represent those of their affiliated organizations, or those of the publisher, the editors and the reviewers. Any product that may be evaluated in this article, or claim that may be made by its manufacturer, is not guaranteed or endorsed by the publisher.
