# Supplementary material for: Mixed oligosaccharides-induced changes in bacterial assembly during cucumber (Cucumis sativus L.) growth
Source: Front Microbiol. 2023 Jul 10;14:1195096. doi: 10.3389/fmicb.2023.1195096 (PMC10364802; doi:10.3389/fmicb.2023.1195096)
Supplement: Supplementary file 6 [file Data_Sheet_1.docx]

Supplementary Material

Mixed oligosaccharides-induced changes in bacterial assembly during the growth of cucumber (*Cucumis sativus* L.)

Qiushui Wang^1, a^, Xin Zhou^3, a^, Yue Liu^1^, Yan Han^2^, Jia Zuo^1^, Jie Deng^1^, Liyan Yuan^1^, Lijuan Gao^1^*, Wenbo Bai^2^*

^1^Institute of Analysis and Testing, Beijing Academy of Science and Technology (Beijing Center for Physical and Chemical Analysis), Beijing, China

^2^Institute of Environment and Sustainable Development in Agriculture, Chinese Academy of Agricultural Sciences, Beijing, China

^3^State Key Laboratory of Mycology, Institute of Microbiology, Chinese Academy of Sciences, Beijing, China

*** Correspondence:**

Wenbo Bai: [baiwenbo@caas.cn](mailto:baiwenbo@caas.cn),

Lijuan Gao: [aglj889@163.com](mailto:aglj889@163.com).

**^a^ These authors contributed equally to this work.**

# Supplementary Methods

**Method S1** Detailed description PCR reaction.

The PCR reaction mixture including 4 μL 5 × Fast Pfu buffer, 2 μL 2.5 mM dNTPs, 0.8 μL each primer (5 μM), 0.4 μL Fast Pfu polymerase, 10 ng of template DNA, and ddH2O to a final volume of 20 µL. PCR amplification cycling conditions were as follows: initial denaturation at 95 ℃ for 3 min, followed by 27 cycles of denaturing at 95 ℃ for 30 s, annealing at 55 ℃ for 30 s and extension at 72 ℃for 45 s, and single extension at 72 ℃ for 10 min, and end at 4 ℃. All samples were amplified in triplicate. The PCR product was extracted from 2% agarose gel and purified using the AxyPrep DNA Gel Extraction Kit (Axygen Biosciences, Union City, CA, USA) according to manufacturer’s instructions and quantified using Quantus™ Fluorometer (Promega, USA).

**Method S2** Chlorophyll meter measurements

A Minolta SPAD 502 chlorophyll meter (Konica Minolta) was used to measure the leaf SPAD values. SPAD readings were measured at the midpoint of the topmost fully developed leaf, and 10 representative plants were randomly selected for the measurements in each experimental plot and averaged (Spaner et al. 2005). The SPAD index was calculated as the ratio of the current SPAD reading to the reference reading from sufficiently fertilized plants (Prost and Jeuffroy 2007).

**Method S3** Antioxidant enzyme activity assay

To conduct activity assays for superoxide dismutase (SOD), peroxidase (POD) and catalase (CAT), samples were extracted using 0.2 m sodium phosphate buffer (pH 6.4) containing 4% PVPP and then centrifuged at 12 000 g for 30 min at 4 °C; the collected supernatant was prepared to measure the enzyme activities. The SOD, POD and CAT activity was measured using commercially available kits (Shanghai Jianglai Industrial Limited by Share Ltd., Shanghai, China) and was expressed as U kg^−1^.

# Supplementary Figures


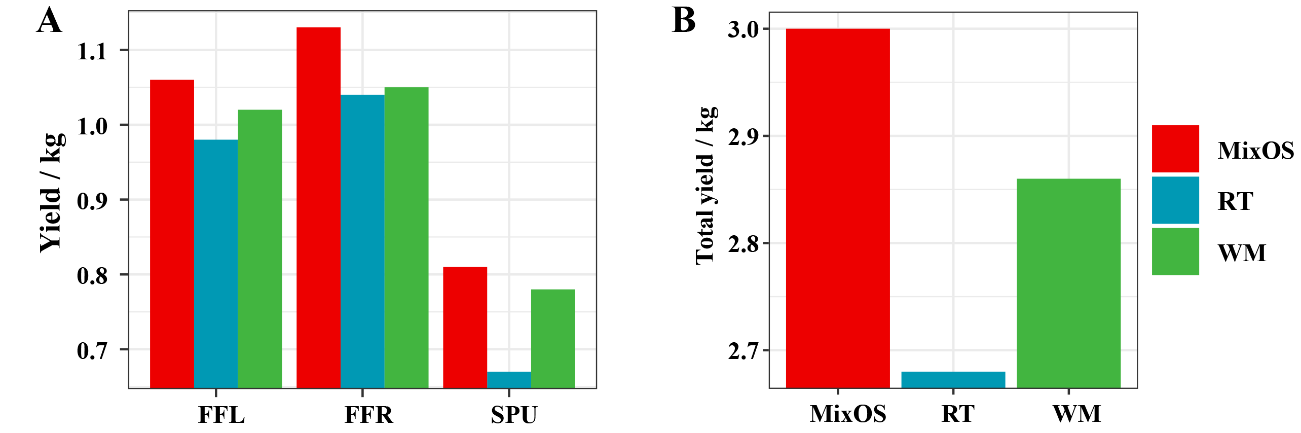


**Figure S1.** Production of cucumbers from different treatment groups in three sampling time. MixOS: Mix Oligosaccharides, RT: Regular Treatment, WM: Water Mimic. FFL: full flowing stage, FFR: full fruit stage, SPU: seedling pulling stage.


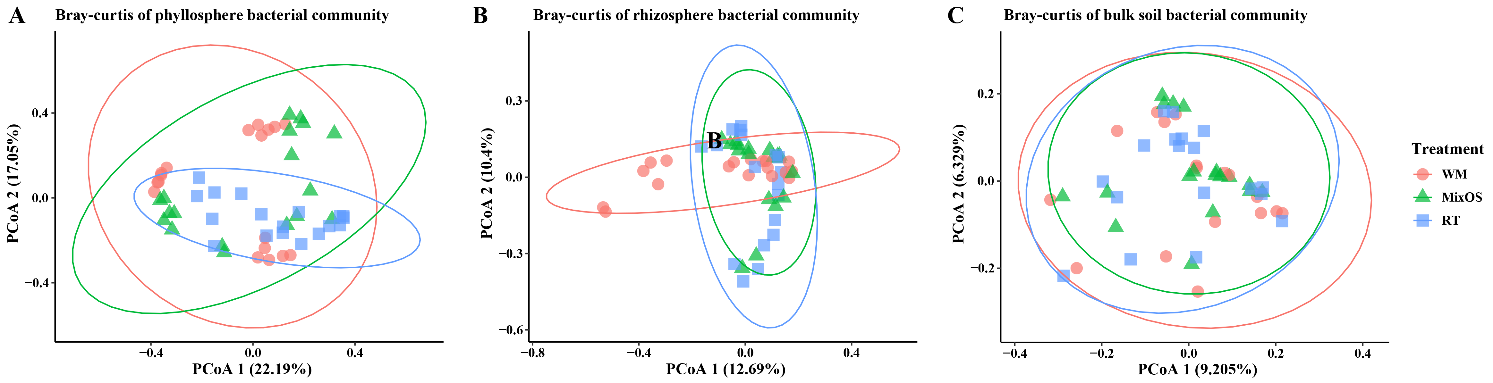


**Figure S2.** The PCoA analysis results for each nich. A: phyllosophere, B: rhizosphere, C: bulk soil.


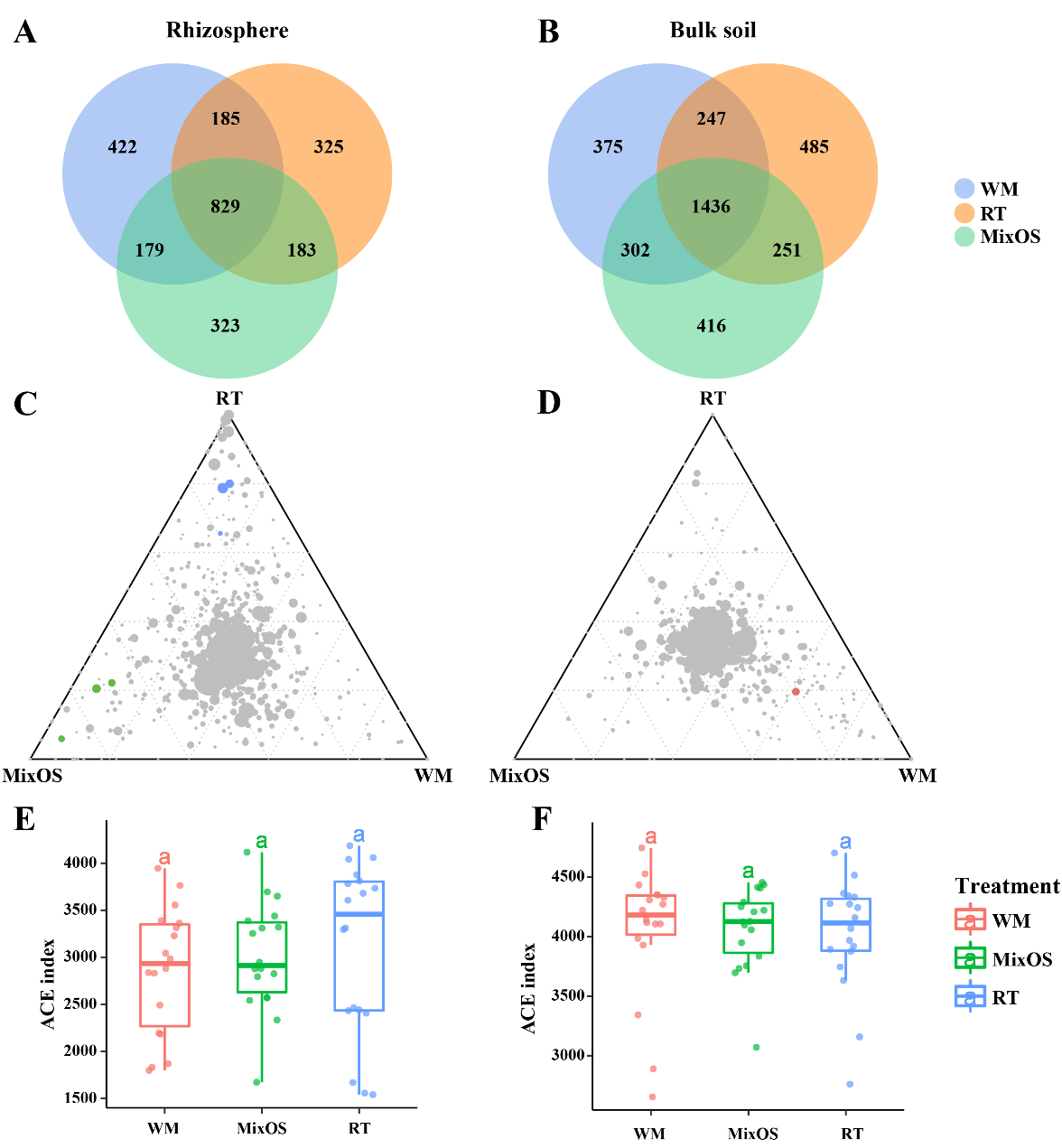


**Figure S3.** (A, B) Veen diagrams of rhizosphere and bulk soil microbiota from different treatments. The ternary plot showed significantly enriched rhizosphere bacterial ASVs (C) and bulk soil bacterial ASVs (D) in WM group (read filled circles), RT group (blue filled circles), and MixOS group (green filled circles), respectively. The gray dots in the center of the ternary plots represent non-significant ASVs shared by all treatment groups. ACE index values of rhizosphere (E) and bulk soil (F) bacterial diversity across different treatments. The ACE index values are colored by different treatments, the line within the box represents the median and bottom, and the top boundaries of the box indicate the 75th and 25th percentile, respectively.


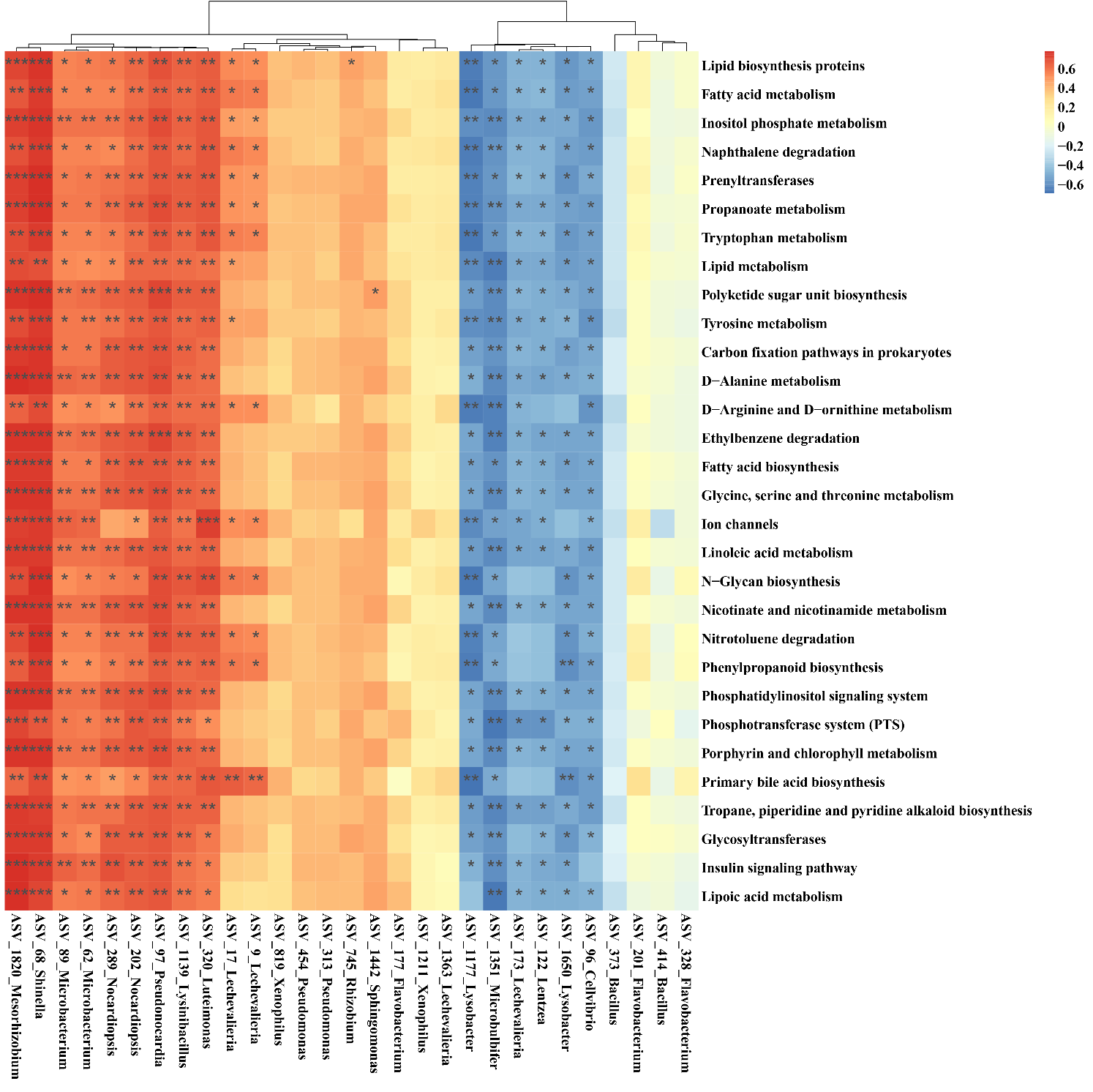


**Figure S4.** The correlation between enriched bacteria in rhizosphere and KEGG pathways calculated by the Spearman correlation coefficients.


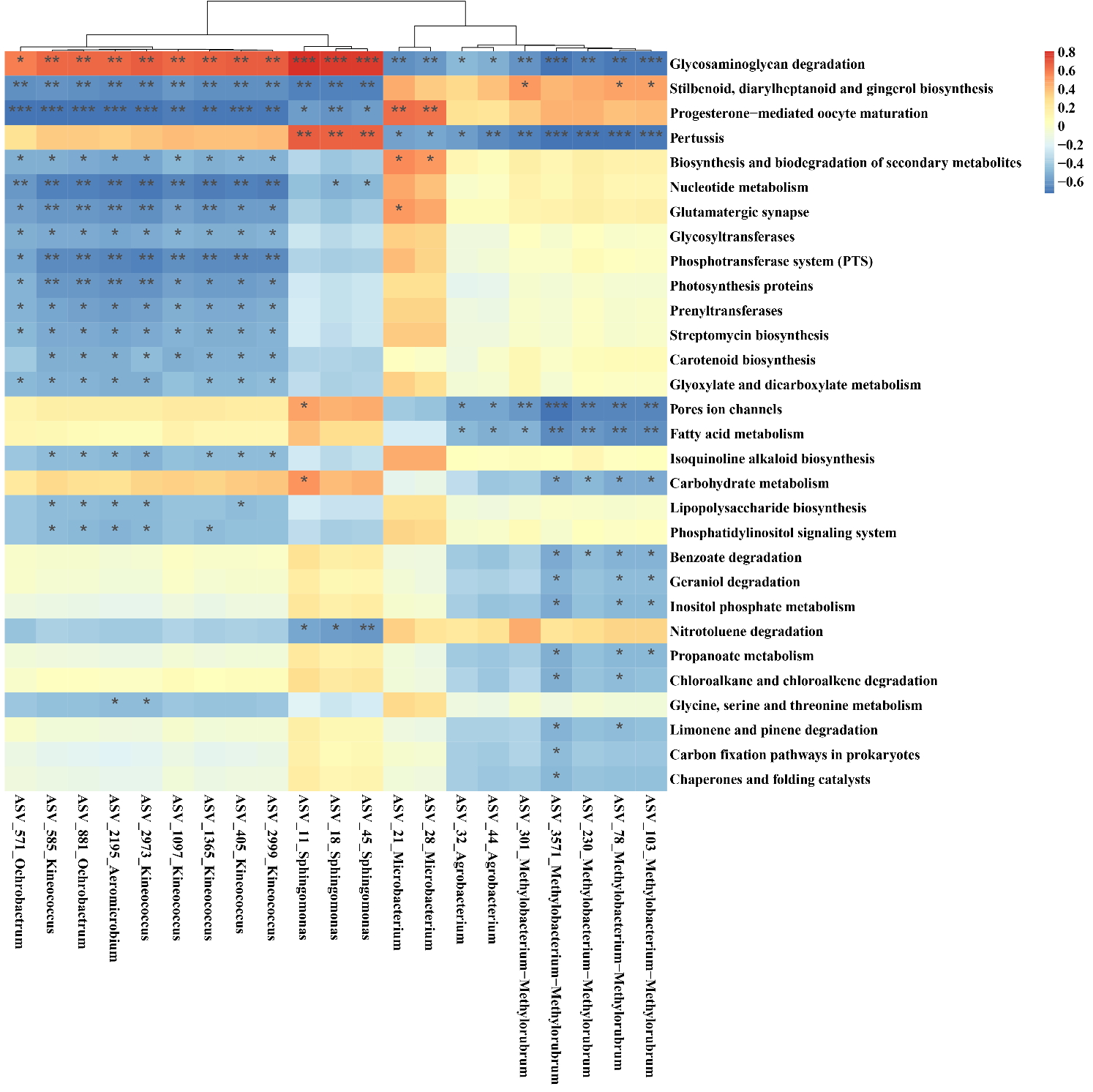


**Figure S5.** The correlation between enriched bacteria in phyllosphere and KEGG pathways calculated by the Spearman correlation coefficients.


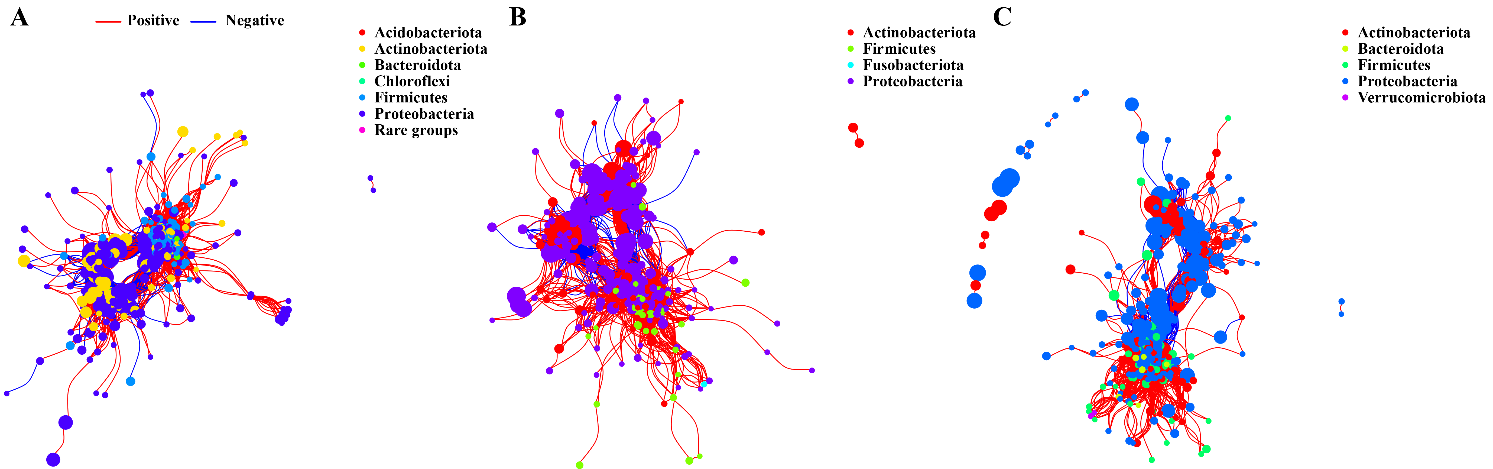


**Figure S6.** Bacterial co-occurrence networks. (A) networks of phyllosphere bacterial communities in WM treatment. (B) networks of phyllosphere bacterial communities in MixOS treatment. (C) networks of phyllosphere bacterial communities in RT treatment.


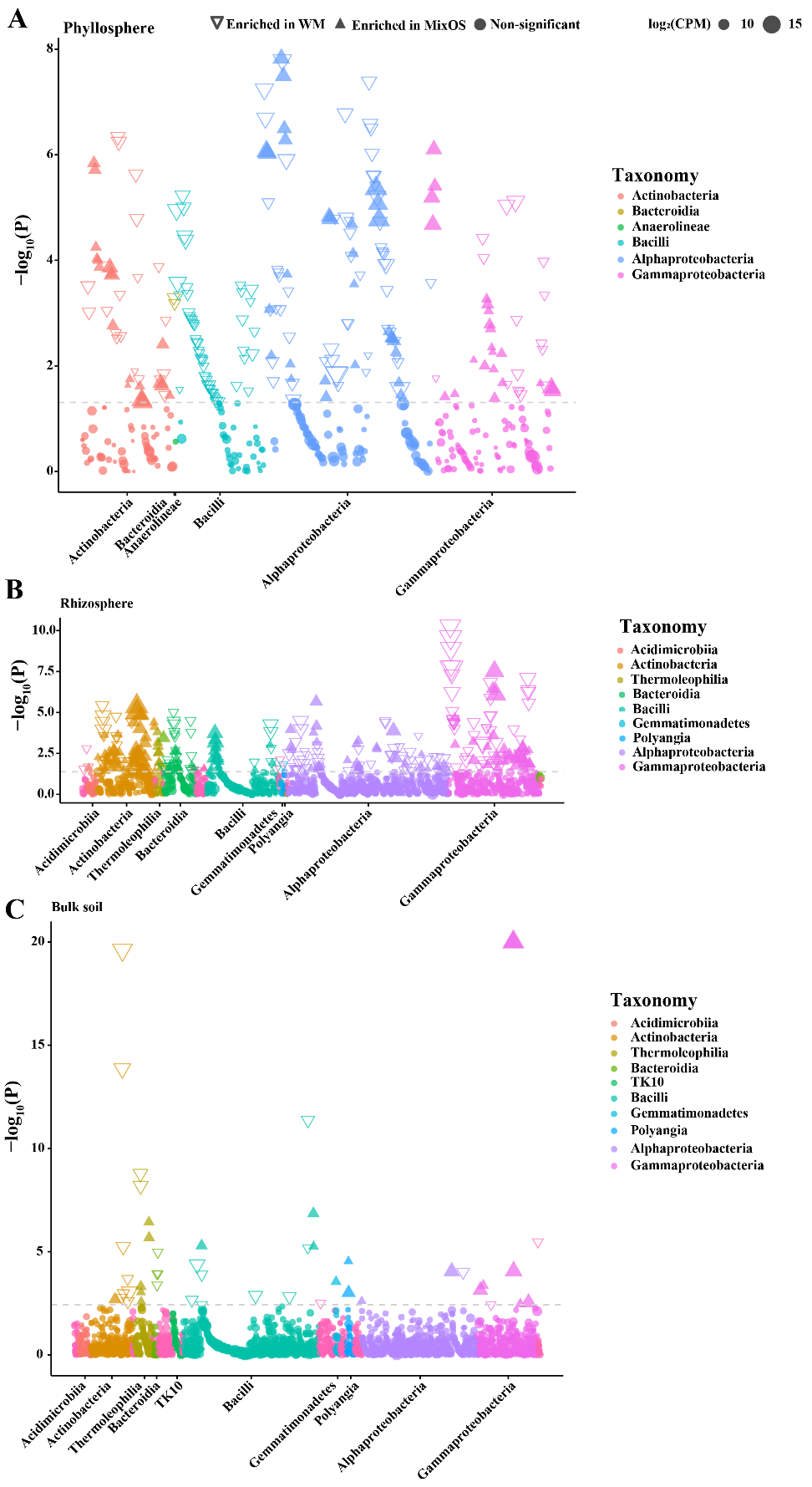


**Figure S7.** Manhattan plots showing MixOS-enriched OTUs with respect to WM-enriched OTUs in (A) phyllosphere, (B) rhizosphere, or (C) bulk soil. OTUs that are significantly enriched. CPM, counts per million.

**Reference**

Prost, Lorène, and Marie-Hélène Jeuffroy. 2007. 'Replacing the nitrogen nutrition index by the chlorophyll meter to assess wheat N status', *Agronomy for Sustainable Development*, 27: 321-30.

Spaner, D., A. G. Todd, A. Navabi, D. B. McKenzie, and L. A. Goonewardene. 2005. 'Can Leaf Chlorophyll Measures at Differing Growth Stages be used as an Indicator of Winter Wheat and Spring Barley Nitrogen Requirements in Eastern Canada?', *Journal of Agronomy and Crop Science*, 191: 393-99.
